# Redetermination of Ba_2_CdTe_3_ from single-crystal X-ray data

**DOI:** 10.1107/S1600536812038974

**Published:** 2012-09-22

**Authors:** Min Yang, Sheng-Qing Xia, Xu-Tang Tao

**Affiliations:** a State Key Laboratory of Crystal Materials, Institute of Crystal Materials, Shandong University, Jinan, Shandong 250100, People’s Republic of China

## Abstract

The previous structure determination of the title compound, dibarium tritelluridocadmate, was based on powder X-ray diffraction data [Wang & DiSalvo (1999 ▶). *J. Solid State Chem.* **148**, 464–467]. In the current redetermination from single-crystal X-ray data, all atoms were refined with anisotropic displacement parameters. The previous structure report is generally confirmed, but with some differences in bond lengths. Ba_2_CdTe_3_ is isotypic with Ba_2_ *MX*_3_ (*M* = Mn, Cd; *X* = S, Se) and features ^1^_∞_[CdTe_2/2_Te_2/1_]^4−^ chains of corner-sharing CdTe_4_ tetra­hedra running parallel [010]. The two Ba^2+^ cations are located between the chains, both within distorted monocapped trigonal–prismatic coordination polyhedra. All atoms in the structure are located on a mirror plane.

## Related literature

For the previous determination of Ba_2_CdTe_3_, see: Wang & DiSalvo (1999 ▶). For isotypic compounds, see: Grey & Steinfink (1971 ▶) for Ba_2_MnS_3_ and Ba_2_MnSe_3_; Iglesias *et al.* (1974 ▶) for Ba_2_CdSe_3_ and Ba_2_CdS_3_.

## Experimental

### 

#### Crystal data

Ba_2_CdTe_3_
*M* *_r_* = 769.88
Orthorhombic, 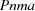

*a* = 9.8405 (2) Å
*b* = 4.7502 (1) Å
*c* = 19.1008 (4) Å
*V* = 892.85 (3) Å^3^
*Z* = 4
Mo *K*α radiation
μ = 20.59 mm^−1^
*T* = 293 K
0.07 × 0.03 × 0.03 mm

#### Data collection

Bruker APEXII CCD diffractometer
Absorption correction: multi-scan (*SADABS*; Bruker, 2005 ▶) *T*_min_ = 0.348, *T*_max_ = 0.627
4014 measured reflections
1098 independent reflections
858 reflections with *I* > 2σ(*I*)
*R*_int_ = 0.031

#### Refinement

*R*[*F* ^2^ > 2σ(*F* ^2^)] = 0.025
*wR*(*F* ^2^) = 0.041
*S* = 0.99
1098 reflections
38 parameters
Δρ_max_ = 1.44 e Å^−3^
Δρ_min_ = −1.27 e Å^−3^

### 

Data collection: *APEX2* (Bruker, 2005 ▶); cell refinement: *SAINT* (Bruker, 2005 ▶); data reduction: *SAINT*; program(s) used to solve structure: *SHELXTL* (Sheldrick, 2008 ▶); program(s) used to refine structure: *SHELXTL*; molecular graphics: *SHELXTL*; software used to prepare material for publication: *SHELXTL*.

## Supplementary Material

Crystal structure: contains datablock(s) I, global. DOI: 10.1107/S1600536812038974/wm2681sup1.cif

Structure factors: contains datablock(s) I. DOI: 10.1107/S1600536812038974/wm2681Isup2.hkl

Additional supplementary materials:  crystallographic information; 3D view; checkCIF report

## Figures and Tables

**Fig. 1.**
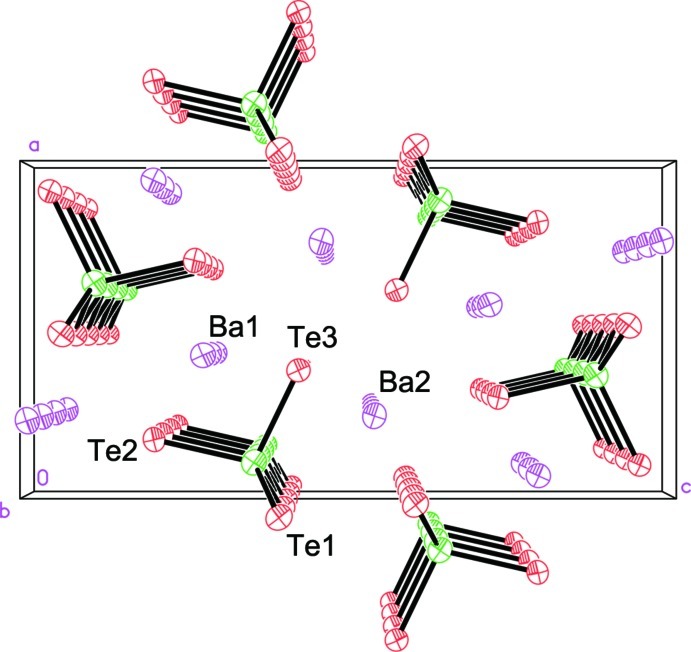
View of the structure of Ba2CdTe3 along the b-axis. The barium, cadmium and tellurium atoms are plotted as purple, green and red ellipsoids, respectively. Ellipsoids are drawn at the 90% probability level.

## supplementary crystallographic information

### Comment

Single crystals of Ba_2_CdTe_3_ were obtained unintentionally from a Bi-flux reactions for exploration of possible new ternary phases in the Ba—Cd—Te system.

The structure of Ba_2_CdTe_3_ is isotypic with Ba_2_Mn*X*_3_ (*X* = S, Se; Grey & Steinfink, 1971) and Ba_2_Cd*X*_3_ (*X* = S, Se; Iglesias *et al.*, 1974). The structural set-up can be described as a packing of polyanionic chains composed of corner-sharing CdTe_4_ tetrahedra. These chains run parallel to [010]; inbetween the chains the two Ba^2+^ cations are located (Fig. 1), both with a coordination number of 7 and surrounded in form of monocapped trigonal-prismatic polyhedra of Te atoms. All atoms are located on a mirror plane *x*, 1/4, *z* (Wyckoff symbol 4*c*).

In comparison with the previous structure model on basis of powder X-ray data (Wang & DiSalvo, 1999), the most important improvement of the current redetermination is reflected in the higher precision of the atomic coordinates and the use of anisotropic displacemenet parameters for all atoms. Although the coordination spheres of Cd and the two Ba atoms can still be described as a distorted CdTe_4_ tetrahedron and two distorted monocapped trigonal BaTe_7_ prisms, respectively, the results of the redetermination indicate some differences in terms of Cd—Te and Ba—Te bond lengths (mean σ for the bond length of the powder model in the range 0.003 Å; 0.0006 for the current model). For example, the longest Ba—Te bonds are 3.6722 (8) and 3.6796 (8) Å for Ba1 and Ba2. The previous powder study revealed distances of 3.638 (5) and 3.500 (5) Å, respectively.

### Experimental

The title compound was synthesized through a high temperature metal flux reaction. All starting elements were handled inside an Argon-filled glove box with controlled oxygen and moisture levels below 0.1 p.p.m.. The reaction conditions were optimized as follows: Ba, Cd, Te and Bi in a molar ratio of 2:1:3:10 were loaded in an alumina crucible, which were subsequently flame-sealed in a fused silica tube. The reactants were heated quickly to 973 K and allowed to dwell at this temperature for 20 h. After a slow cooling process down to 773 K at a rate of 5 K/h and the removal of the Bi flux by centrifugation, high-quality single crystals of Ba_2_CdTe_3_ were obtained.

### Refinement

The full occupancies for all sites were verified by freeing the site occupation factor for an individual atom, while other remaining parameters were kept fixed. This proved that all positions are fully occupied with corresponding deviations from full occupancy within 3σ. The residual electron densities show a maximum peak of 1.44 e/Å^3^ and a minimum hole of -1.27 e/Å^3^, which are 0.86 and 0.81 Å from Te3 and Te2, respectively.

### Crystal data

**Table:**

Ba_2_CdTe_3_	*F*(000) = 1264
*M**_r_* = 769.88	*D*_x_ = 5.727 Mg m^−^^3^
Orthorhombic, *P**n**m**a*	Mo *K*α radiation, λ = 0.71073 Å
Hall symbol: -P 2ac 2n	Cell parameters from 1715 reflections
*a* = 9.8405 (2) Å	θ = 3.0–28.2°
*b* = 4.7502 (1) Å	µ = 20.59 mm^−^^1^
*c* = 19.1008 (4) Å	*T* = 293 K
*V* = 892.85 (3) Å^3^	Needle, red
*Z* = 4	0.07 × 0.03 × 0.03 mm

### Data collection

**Table:**

Bruker APEXII CCD diffractometer	1098 independent reflections
Radiation source: fine-focus sealed tube	858 reflections with *I* > 2σ(*I*)
Graphite monochromator	*R*_int_ = 0.031
φ and ω scans	θ_max_ = 27.1°, θ_min_ = 2.1°
Absorption correction: multi-scan (*SADABS*; Bruker, 2005)	*h* = −12→9
*T*_min_ = 0.348, *T*_max_ = 0.627	*k* = −3→6
4014 measured reflections	*l* = −24→15

### Refinement

**Table:**

Refinement on *F*^2^	Primary atom site location: structure-invariant direct methods
Least-squares matrix: full	Secondary atom site location: difference Fourier map
*R*[*F*^2^ > 2σ(*F*^2^)] = 0.025	*w* = 1/[σ^2^(*F*_o_^2^) + (0.0142*P*)^2^] where *P* = (*F*_o_^2^ + 2*F*_c_^2^)/3
*wR*(*F*^2^) = 0.041	(Δ/σ)_max_ = 0.001
*S* = 0.99	Δρ_max_ = 1.44 e Å^−^^3^
1098 reflections	Δρ_min_ = −1.27 e Å^−^^3^
38 parameters	Extinction correction: *SHELXTL* (Sheldrick, 2008), Fc^*^=kFc[1+0.001xFc^2^λ^3^/sin(2θ)]^-1/4^
0 restraints	Extinction coefficient: 0.00112 (6)

### Special details

**Table:**

Geometry. All e.s.d.'s (except the e.s.d. in the dihedral angle between two l.s. planes) are estimated using the full covariance matrix. The cell e.s.d.'s are taken into account individually in the estimation of e.s.d.'s in distances, angles and torsion angles; correlations between e.s.d.'s in cell parameters are only used when they are defined by crystal symmetry. An approximate (isotropic) treatment of cell e.s.d.'s is used for estimating e.s.d.'s involving l.s. planes.
Refinement. Refinement of *F*^2^ against ALL reflections. The weighted *R*-factor *wR* and goodness of fit *S* are based on *F*^2^, conventional *R*-factors *R* are based on *F*, with *F* set to zero for negative *F*^2^. The threshold expression of *F*^2^ > σ(*F*^2^) is used only for calculating *R*-factors(gt) *etc*. and is not relevant to the choice of reflections for refinement. *R*-factors based on *F*^2^ are statistically about twice as large as those based on *F*, and *R*- factors based on ALL data will be even larger.

### Fractional atomic coordinates and isotropic or equivalent isotropic displacement parameters (Å^2^)

**Table:**

	*x*	*y*	*z*	*U*_iso_*/*U*_eq_	
Ba1	0.07317 (6)	0.2500	0.78653 (3)	0.01857 (16)	
Ba2	0.24597 (6)	0.2500	0.03873 (3)	0.01918 (16)	
Cd1	0.12996 (7)	0.2500	0.36470 (3)	0.01947 (18)	
Te1	0.01147 (6)	0.2500	0.59692 (3)	0.01802 (17)	
Te2	0.19335 (6)	0.2500	0.22108 (3)	0.01748 (16)	
Te3	0.38656 (6)	0.2500	0.42865 (3)	0.01745 (17)	

### Atomic displacement parameters (Å^2^)

**Table:**

	*U*^11^	*U*^22^	*U*^33^	*U*^12^	*U*^13^	*U*^23^
Ba1	0.0190 (3)	0.0171 (3)	0.0196 (4)	0.000	−0.0003 (3)	0.000
Ba2	0.0209 (3)	0.0191 (3)	0.0175 (3)	0.000	−0.0013 (3)	0.000
Cd1	0.0197 (4)	0.0201 (4)	0.0186 (4)	0.000	0.0014 (3)	0.000
Te1	0.0187 (4)	0.0154 (3)	0.0200 (4)	0.000	0.0024 (3)	0.000
Te2	0.0178 (4)	0.0203 (3)	0.0144 (4)	0.000	−0.0008 (3)	0.000
Te3	0.0158 (4)	0.0200 (3)	0.0165 (4)	0.000	0.0004 (3)	0.000

### Geometric parameters (Å, º)

**Table:**

Ba1—Te2^i^	3.5331 (6)	Cd1—Te1^iii^	2.8488 (5)
Ba1—Te2^ii^	3.5331 (6)	Cd1—Te1^iv^	2.8488 (5)
Ba1—Te2^iii^	3.5413 (6)	Te1—Cd1^iii^	2.8488 (5)
Ba1—Te2^iv^	3.5413 (6)	Te1—Cd1^iv^	2.8488 (5)
Ba1—Te3^i^	3.6287 (6)	Te1—Ba2^i^	3.5459 (6)
Ba1—Te3^ii^	3.6287 (6)	Te1—Ba2^ii^	3.5459 (6)
Ba1—Te1	3.6722 (8)	Te1—Ba2^vii^	3.6796 (8)
Ba2—Te3^v^	3.4297 (6)	Te2—Ba1^vi^	3.5331 (6)
Ba2—Te3^vi^	3.4297 (6)	Te2—Ba1^v^	3.5331 (6)
Ba2—Te2	3.5213 (8)	Te2—Ba1^iii^	3.5413 (6)
Ba2—Te1^vi^	3.5459 (6)	Te2—Ba1^iv^	3.5413 (6)
Ba2—Te1^v^	3.5459 (6)	Te3—Ba2^i^	3.4297 (6)
Ba2—Te3^vii^	3.5913 (8)	Te3—Ba2^ii^	3.4297 (6)
Ba2—Te1^viii^	3.6796 (8)	Te3—Ba2^viii^	3.5913 (8)
Cd1—Te3	2.8050 (9)	Te3—Ba1^v^	3.6287 (6)
Cd1—Te2	2.8133 (9)	Te3—Ba1^vi^	3.6287 (6)
			
Te2^i^—Ba1—Te2^ii^	84.480 (18)	Te1^iii^—Cd1—Te1^iv^	112.97 (3)
Te2^i^—Ba1—Te2^iii^	156.629 (18)	Cd1^iii^—Te1—Cd1^iv^	112.97 (3)
Te2^ii^—Ba1—Te2^iii^	90.930 (5)	Cd1^iii^—Te1—Ba2^i^	165.46 (2)
Te2^i^—Ba1—Te2^iv^	90.930 (5)	Cd1^iv^—Te1—Ba2^i^	81.436 (14)
Te2^ii^—Ba1—Te2^iv^	156.629 (18)	Cd1^iii^—Te1—Ba2^ii^	81.436 (14)
Te2^iii^—Ba1—Te2^iv^	84.241 (18)	Cd1^iv^—Te1—Ba2^ii^	165.46 (2)
Te2^i^—Ba1—Te3^i^	75.741 (13)	Ba2^i^—Te1—Ba2^ii^	84.104 (18)
Te2^ii^—Ba1—Te3^i^	129.32 (2)	Cd1^iii^—Te1—Ba1	80.038 (19)
Te2^iii^—Ba1—Te3^i^	123.41 (2)	Cd1^iv^—Te1—Ba1	80.038 (19)
Te2^iv^—Ba1—Te3^i^	70.888 (14)	Ba2^i^—Te1—Ba1	101.413 (18)
Te2^i^—Ba1—Te3^ii^	129.32 (2)	Ba2^ii^—Te1—Ba1	101.413 (18)
Te2^ii^—Ba1—Te3^ii^	75.741 (13)	Cd1^iii^—Te1—Ba2^vii^	80.46 (2)
Te2^iii^—Ba1—Te3^ii^	70.888 (14)	Cd1^iv^—Te1—Ba2^vii^	80.46 (2)
Te2^iv^—Ba1—Te3^ii^	123.41 (2)	Ba2^i^—Te1—Ba2^vii^	104.904 (17)
Te3^i^—Ba1—Te3^ii^	81.769 (17)	Ba2^ii^—Te1—Ba2^vii^	104.904 (17)
Te2^i^—Ba1—Te1	76.026 (15)	Ba1—Te1—Ba2^vii^	144.28 (2)
Te2^ii^—Ba1—Te1	76.026 (15)	Cd1—Te2—Ba2	175.65 (3)
Te2^iii^—Ba1—Te1	80.624 (16)	Cd1—Te2—Ba1^vi^	78.412 (18)
Te2^iv^—Ba1—Te1	80.624 (16)	Ba2—Te2—Ba1^vi^	104.737 (17)
Te3^i^—Ba1—Te1	139.101 (8)	Cd1—Te2—Ba1^v^	78.412 (18)
Te3^ii^—Ba1—Te1	139.101 (8)	Ba2—Te2—Ba1^v^	104.737 (17)
Te3^v^—Ba2—Te3^vi^	87.658 (19)	Ba1^vi^—Te2—Ba1^v^	84.480 (18)
Te3^v^—Ba2—Te2	123.399 (16)	Cd1—Te2—Ba1^iii^	82.866 (18)
Te3^vi^—Ba2—Te2	123.399 (16)	Ba2—Te2—Ba1^iii^	93.918 (17)
Te3^v^—Ba2—Te1^vi^	155.78 (2)	Ba1^vi^—Te2—Ba1^iii^	161.261 (19)
Te3^vi^—Ba2—Te1^vi^	89.100 (12)	Ba1^v^—Te2—Ba1^iii^	92.594 (5)
Te2—Ba2—Te1^vi^	77.814 (17)	Cd1—Te2—Ba1^iv^	82.866 (18)
Te3^v^—Ba2—Te1^v^	89.100 (12)	Ba2—Te2—Ba1^iv^	93.918 (17)
Te3^vi^—Ba2—Te1^v^	155.78 (2)	Ba1^vi^—Te2—Ba1^iv^	92.594 (5)
Te2—Ba2—Te1^v^	77.814 (17)	Ba1^v^—Te2—Ba1^iv^	161.261 (19)
Te1^vi^—Ba2—Te1^v^	84.104 (18)	Ba1^iii^—Te2—Ba1^iv^	84.240 (18)
Te3^v^—Ba2—Te3^vii^	74.447 (16)	Cd1—Te3—Ba2^i^	85.679 (19)
Te3^vi^—Ba2—Te3^vii^	74.447 (16)	Cd1—Te3—Ba2^ii^	85.679 (19)
Te2—Ba2—Te3^vii^	71.552 (17)	Ba2^i^—Te3—Ba2^ii^	87.658 (19)
Te1^vi^—Ba2—Te3^vii^	127.482 (15)	Cd1—Te3—Ba2^viii^	164.18 (3)
Te1^v^—Ba2—Te3^vii^	127.482 (15)	Ba2^i^—Te3—Ba2^viii^	105.553 (16)
Te3^v^—Ba2—Te1^viii^	80.695 (16)	Ba2^ii^—Te3—Ba2^viii^	105.553 (16)
Te3^vi^—Ba2—Te1^viii^	80.695 (16)	Cd1—Te3—Ba1^v^	76.852 (18)
Te2—Ba2—Te1^viii^	143.22 (2)	Ba2^i^—Te3—Ba1^v^	162.44 (2)
Te1^vi^—Ba2—Te1^viii^	75.096 (17)	Ba2^ii^—Te3—Ba1^v^	92.687 (10)
Te1^v^—Ba2—Te1^viii^	75.096 (17)	Ba2^viii^—Te3—Ba1^v^	91.275 (17)
Te3^vii^—Ba2—Te1^viii^	145.23 (2)	Cd1—Te3—Ba1^vi^	76.852 (18)
Te3—Cd1—Te2	103.01 (3)	Ba2^i^—Te3—Ba1^vi^	92.687 (10)
Te3—Cd1—Te1^iii^	109.13 (2)	Ba2^ii^—Te3—Ba1^vi^	162.44 (2)
Te2—Cd1—Te1^iii^	111.05 (2)	Ba2^viii^—Te3—Ba1^vi^	91.275 (17)
Te3—Cd1—Te1^iv^	109.13 (2)	Ba1^v^—Te3—Ba1^vi^	81.769 (17)
Te2—Cd1—Te1^iv^	111.05 (2)		

Symmetry codes: (i) −*x*+1/2, −*y*+1, *z*+1/2; (ii) −*x*+1/2, −*y*, *z*+1/2; (iii) −*x*, −*y*, −*z*+1; (iv) −*x*, −*y*+1, −*z*+1; (v) −*x*+1/2, −*y*, *z*−1/2; (vi) −*x*+1/2, −*y*+1, *z*−1/2; (vii) *x*−1/2, *y*, −*z*+1/2; (viii) *x*+1/2, *y*, −*z*+1/2.
